# Learning from agriculture: understanding low-dose antimicrobials as drivers of resistome expansion

**DOI:** 10.3389/fmicb.2014.00284

**Published:** 2014-06-10

**Authors:** Yaqi You, Ellen K. Silbergeld

**Affiliations:** Department of Environmental Health Sciences, Johns Hopkins Bloomberg School of Public Health, Johns Hopkins UniversityBaltimore, MD, USA

**Keywords:** agriculture, antimicrobials, metals, microbiome, resistome, mobilome, environmental pollution

## Abstract

Antimicrobial resistance is a growing public health challenge worldwide, with agricultural use of antimicrobials being one major contributor to the emergence and dissemination of antimicrobial resistance (AMR). Globally, most antimicrobials are used in industrial food animal production, a major context for microbiomes encountering low-doses or subtherapeutic-levels of antimicrobial agents from all mechanistic classes. This modern practice exerts broad eco-evolutionary effects on the gut microbiome of food animals, which is subsequently transferred to animal waste. This waste contains complex constituents that are challenging to treat, including AMR determinants and low-dose antimicrobials. Unconfined storage or land deposition of a large volume of animal waste causes its wide contact with the environment and drives the expansion of the environmental resistome through mobilome facilitated horizontal genet transfer. The expanded environmental resistome, which encompasses both natural constituents and anthropogenic inputs, can persist under multiple stressors from agriculture and may re-enter humans, thus posing a public health risk to humans. For these reasons, this review focuses on agricultural antimicrobial use as a laboratory for understanding low-dose antimicrobials as drivers of resistome expansion, briefly summarizes current knowledge on this topic, highlights the importance of research specifically on environmental microbial ecosystems considering AMR as environmental pollution, and calls attention to the needs for longitudinal studies at the systems level.

## Introduction

The intensive production of food animals is a major context for microbiomes encountering low-doses or subtherapeutic-levels of diverse classes of antimicrobial agents. For that reason, this review focuses on food animal production as an important laboratory for understanding the eco- evolutionary (interactions and intersection of ecology and evolutionary biology) mechanisms involved in bacterial responses to low-dose, sub-therapeutic pressures associated with antimicrobials. A majority of agricultural antimicrobials are used as feed additives for growth promotion in livestock and poultry (Silbergeld et al., 2008). This use began in the 1940s in the US, soon after the initiation of large-scale production of these drugs for clinical medicine. From the first approvals to the present, the concentrations of growth promoting antimicrobials (GPAs) in feeds have been stipulated by FDA regulation to deliver sub-therapeutic doses. Redefining GPA use as “therapeutic,” “non-therapeutic,” or “prophylactic” to comply with regulations in some countries and guidance by the US FDA does not change this condition from the microbial perspective.

This paper will not discuss the hypothesized mechanisms by which GPAs are asserted to increase growth and feed efficiency, since a recent large study by the Perdue Company reported that there were very small or non-significant differences in these outcomes among poultry flocks consuming feeds with or without GPAs (Engster et al., 2002; Graham et al., 2007). In contrast to earlier studies, including those at Lederle (Stokstad and Jukes, 1958-1959), this is the only study conducted under empirical conditions in poultry production over the lifespan of the animals.

GPA use employs agents from every mechanistic class and currently exceeds all clinical uses in terms of the proportion of total antimicrobial production in the US (Silbergeld et al., 2008; FDA, 2011) and, until recently, in the EU (Teuber, 2001). Information from other regions is not generally available, but given the global expansion of poultry and livestock production using methods similar to those first developed in the US (Graham et al., 2008), it is likely that global use of antimicrobials in animal feeds is also significant (see Arriola, 2011 for study of Peru). As a result, GPAs have had important impacts on selection and dissemination of antimicrobial resistance (AMR) worldwide through the food supply and environmental releases. Moreover, because GPAs are utilized most commonly as mixtures in animal feeds, the gut microbiome of poultry or livestock is exposed to multiple pressures acting on a range of molecular mechanisms associated with resistance development (Davis et al., 2011).

From early in the history of GPA use, it was recognized that the gut microbiome of poultry was responding to selection for resistance to drugs in feeds. Jukes acknowledged this as a truism, but discounted any potential risks for human health (1972). With more concern, Starr and Reynolds (1951) reported that *Escherichia coli* isolated from the gut microflora of poultry flocks fed with streptomycin as a GPA were resistant to streptomycin, as compared to isolates from unexposed flocks. Since that time, numerous studies have documented associations between GPA use and temporal and geographic trends in AMR prevalence in animal wastes, food products, and human populations (documented most completely in studies in Denmark e.g., Aarestrup et al., 2001; Wegener, 2003, also see review by Silbergeld et al., 2008).

The use of molecular, genomic, and metagenomic methods to track AMR genes and AMR strains from food animal production has increased the strength of the evidence on this connection. These methods have also clarified implications of intensive food animal production for human health, demonstrating that the microbiomes of livestock and poultry are reservoirs for AMR pathogens and that resistance determinants can be transferred from these microbiomes to the environment and eventually to humans (Hammerum, 2012). This has been most recently demonstrated for livestock specific strains of methicillin resistant *Staphylococcus aureus* (van Loo et al., 2007; Waters et al., 2011; Price et al., 2012) and extraintestinal pathogenic *E. coli*, especially those phylogroups associated with urinary tract infection in humans (Jakobsen et al., 2010). Smet et al. (2011) reported that a plasmid carrying the *bla*_TEM−52_ gene encoding ESBL (extended-spectrum β-lactamase) could be transferred from a poultry strain of *E. coli* into human *E. coli* under simulated human caecal conditions. The same ESBL genes (including *bla*_TEM−52_), ESBL-encoding plasmids and ESBL-producing *E. coli* strains have been observed in poultry, chicken meat and humans (Leverstein-van Hall et al., 2011).

This paper briefly summarizes current knowledge on this topic and highlights the importance of research specifically on environmental microbial ecosystems for understanding the evolution and persistence of resistance associated with GPA use in agriculture. This reflects the fact that many events related to the emergence of AMR in agricultural settings occur in the context of interactions between the gut microbiome of food animals, such as chickens and pigs, and environmental microbiomes in those ecological niches impacted by disposal of animal wastes. These wastes are normally not treated prior to disposal and often contain AMR bacteria and transmissible genetic elements assembling resistance genes, as well as residual antimicrobials and their degradation products.

## Current knowledge

Over the past three decades, research has largely investigated the agricultural setting for AMR emergence primarily for the purpose of understanding the origin of AMR in food borne pathogens. Relatively recently, non-food pathways and the role of the environment have attracted increasing research attention. However, incorporating this knowledge into studies of microbial ecology and evolution as well as into studies of disease outbreaks and attributable risk of infection by drug resistant bacteria is still limited (Ashbolt et al., 2013).

## Eco-evolutionary consequences of industrial food animal production on the gut microbiome of food animals

Current methods and conditions in industrial food animal production are quite different from traditional agronomy in terms of intensity and density as well as the use of GPAs (Silbergeld et al., 2008). These changes are likely to have affected animal gut microbiomes. Looft et al. (2012), using phylogenetic and metagenomic approaches, found that 14 days' exposure to subtherapeutic doses of chlortetracycline, sulfamethazine, and penicillin induced shifts in gut microbiota of pigs. These changes included an increase in the prevalence of *Proteobacteria* (primarily in *E. coli* species) and in the abundance and diversity of AMR genes specific to and beyond those used GPAs, as well as selection for other genes related to gene transfer, virulence, energy production, and energy conversion. Population shifts were also observed in the gut microbiome of pigs receiving the GPA tylosin (Kim et al., 2012). Using metagenomic pyrosequencing, Danzeisen et al. (2011) reported that the GPA mixtures of virginiamycin/monensin or tylosin/monensin enriched *E. coli* populations in the chicken cecal microbiome, along with genes encoding transport systems, type I fimbriae and type IV conjugative secretion systems. That study did not detect significant differences in AMR gene occurrence between GPA treatment and control groups, which may not represent the commercial poultry production conditions. While AMR genes are present in the gut microbiome of free-range chickens (Zhou et al., 2012), a diverse pool of AMR genes is found in conventionally raised chickens (Qu et al., 2008; Zhou et al., 2012).

The mobilome (Siefert, 2009), a collection of all mobile genetic elements (MGEs), is a functional component of the microbiome, and plays an essential role in microbial ecology and evolution by facilitating horizontal gene transfer among microorganisms (Frost et al., 2005; Gillings, 2013). There are some reports on impacts of GPAs on the animal gut mobilome (Danzeisen et al., 2011; Looft et al., 2012), but studies at the systems level are still rare. Antimicrobials, in addition to enriching preexisting AMR genotypes and phenotypes and providing pressure for evolutionary selection for *de novo* mutations that favor survival (Gullberg et al., 2011), also induce horizontal transfer of MGEs through mechanisms such as bacterial SOS response (Beaber et al., 2003) and translation attenuation (Wozniak and Waldor, 2010). Some recent studies have identified the contributions of GPAs to phage-mediated horizontal transfer of AMR genes in the animal gut microbiome. Allen et al. (2011) reported that the GPA mixture Aureomix 500 used in swine feed (containing chlortetracycline, sulfamethazine and penicillin) led to significant population shifts in both phage and bacterial communities, as well as induction of prophages (one type of MGEs) in swine fecal microbiomes. The altered phage metagenomes harbored multiple AMR genes including genes for multidrug resistance. Bearson et al. (2014) reported that the GPA carbadox induced phage mediated transfer of virulence and resistance genes in *Salmonella enterica* serovar Typhimurium, a human foodborne pathogen that frequently colonizes swine. More investigation is needed for other components of the animal gut mobilome, such as the plasmidome (Kav et al., 2012), before we can fully understand impacts of GPAs on the entire mobilome within the animal gut microbiome.

## Animal wastes: The connector between the gut microbiome of food animals and the environment

**Figure 1:**
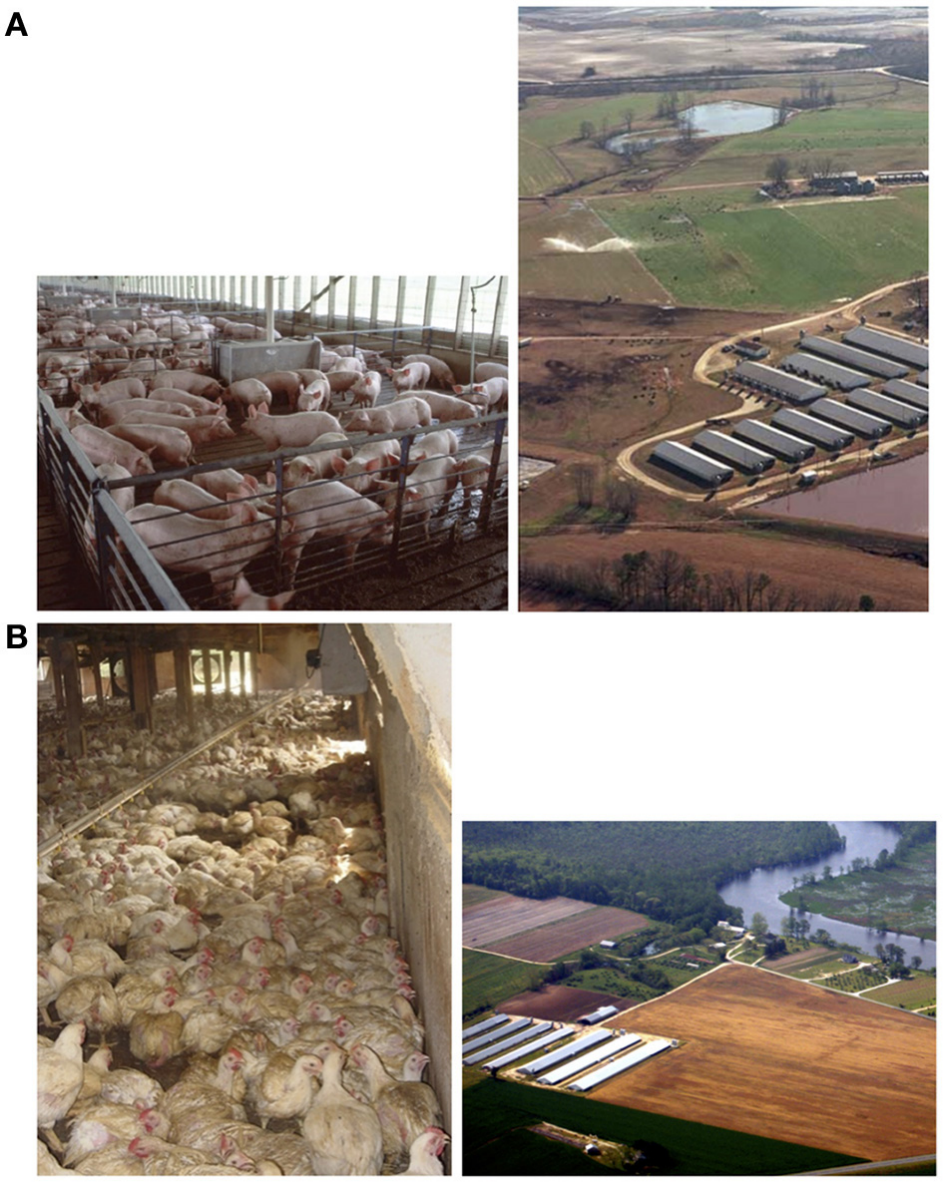
**(A)** (left) Pigs in confinement house (photo USDA). Note slatted floor; wastes (including excreta and spilled feed) accumulate on this surface and are periodically washed down into a cesspit below the building. (right) A view of a swine production operation showing open cesspits that collect drainage from animal houses; disposal of these wastes involves spraying of liquids (photo S Wing). **(B)** (left) Chickens in a Maryland poultry house. Flocks are housed directly on litter, which contains excreta (as evident from the birds) as well as spilled feed. Litter is removed infrequently from poultry houses (photo J Graham). (right) The lower Pocomoke River, with poultry houses and land disposal of poultry waste (source: Integration and Application Network, University of Maryland Center for Environmental Sciences).

The gut microbiome of poultry and livestock raised in confinement is transferred into animal excreta, which include the resistome and the mobilome. Also present in animal excreta are unmetabolized GPAs (Kumar et al., 2005; Sarmah et al., 2006) and active metabolites. Thus, the first nexus in the environmental pathway of the dissemination of animal husbandry originated AMR is poultry house litter or cesspits that collect wastes in swine barns (see Figure 1).

Within a confinement house, wastes (solid and liquid) accumulate inputs from animal excreta and other residues like spilled feed containing GPAs into a waste microbiome over the lifetime of a flock or a herd, and often for a longer period when poultry houses are not routinely cleaned between each flock or when septic impoundments are not emptied between herds (Volkova et al., 2009). During this period, the microbiomes of chickens and house litter interact and exchange organisms and resistance genes (Cressman et al., 2010; Shanmugasundaram et al., 2011). Eco-evolutionary events in the litter microbiome have not been carefully studied. But poultry litter is known to be a reservoir of AMR bacteria and resistance determinants, resistance-encoding MGEs and residual antimicrobials (Nandi et al., 2004; Furtula et al., 2009; Graham et al., 2009a; Cheng et al., 2013).

Environmental dissemination of the food-animal-associated microbiome and resistome occurs through waste holding and disposal. Food animal wastes are first released to the environment through on site storage (usually in open sheds for poultry or open impoundments for swine) and then discharged into the environment more broadly by land application as shown in Figure 1. The sites of land disposal may be close to or very distant from the sites of poultry or swine production (Leibler et al., 2009). Once released into the external environment either by unconfined storage or by deposition on land, the microbial and chemical constituents of animal wastes can be widely dispersed mainly through dust, air, and water movement, as well as by animal movement (flies, rodents, and wild birds) (Graham et al., 2009b).

Because of the intensity of food animal production in the US and other countries, this constitutes a major source of all gut microbiota being transferred to the environment (Silbergeld et al., 2008). Unlike human biosolids, no treatment is required prior to discharge of animal wastes, which are almost entirely (>90%) disposed of onto land (Graham and Nachman, 2010). Storage of poultry house litter or of swine waste, without specific composting procedures, does not reduce the burden of pathogens and AMR determinants (Gerba and Smith, 2005; Graham et al., 2009a). More intensive composting, though more effective in attenuating microbial loadings in wastes, does not significantly reduce loadings of AMR genes (Storteboom et al., 2007). Even multiple treatment lagoons are unable to completely remove AMR genes (McKinney et al., 2010).

**Figure 2:**
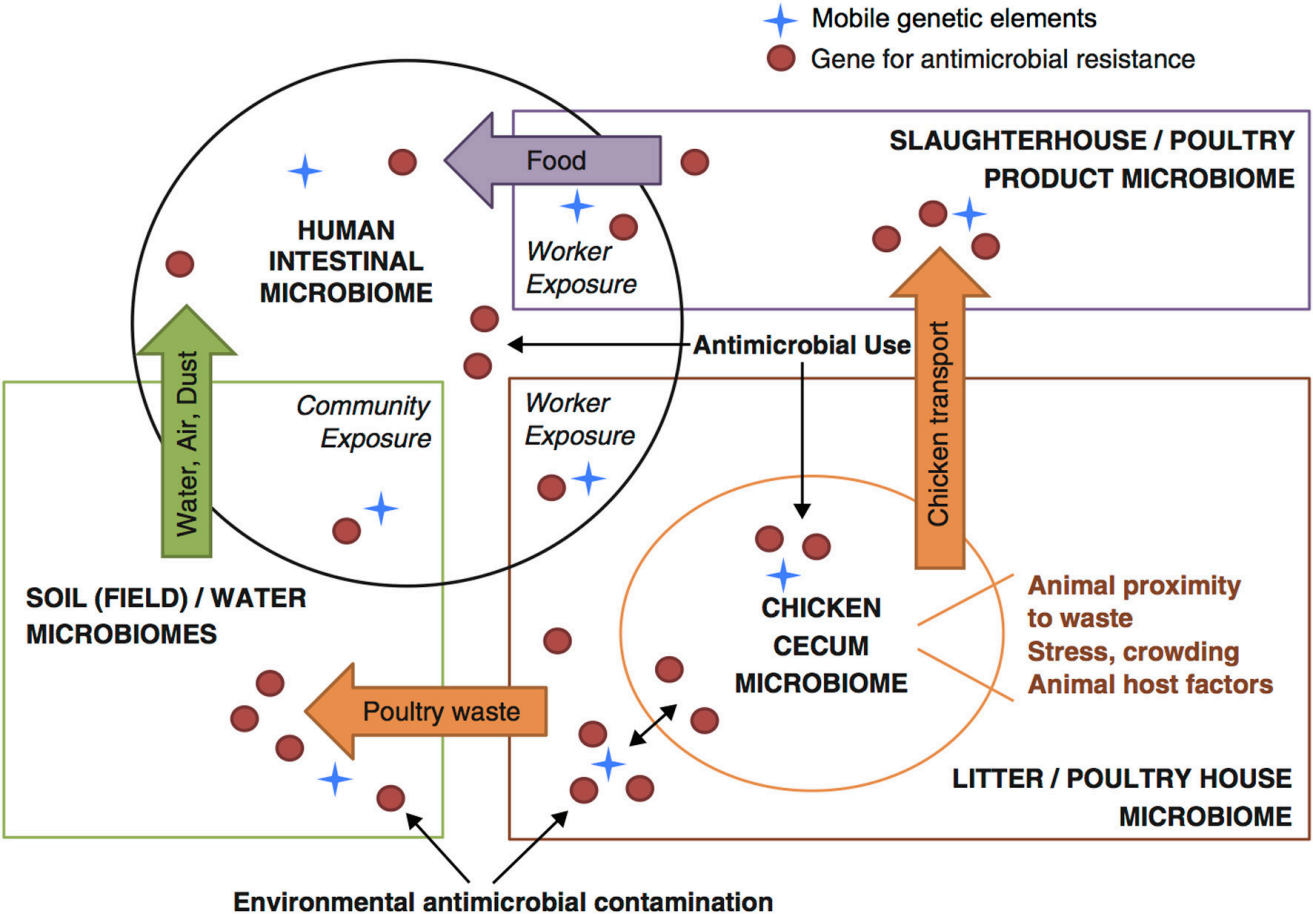
**Conceptual framework for understanding flow of resistance genes and mobile genetic elements across microbiomes within food animals, the environment, and human populations (based on Davis et al., 2011)**.

For these characteristics of animal wastes—complex constituents, large volume, insufficient waste management, and broad contact with the environment—the appropriate focus for understanding the emergence and dissemination of AMR driven by use of subtherapeutic GPAs in agriculture is in the interactions between animal wastes and the environment. Intensive animal production operations and associated environmental compartments have been characterized as “genetic reactors” where new genotypes and phenotypes of resistance can evolve through genetic exchange and recombination, and re-enter humans and animals (Baquero et al., 2008). We conceive of a series of dynamic interactions between environmental microbiomes and the altered gut microbiome of poultry flocks that is represented within poultry house waste, which in turn integrates microbial loadings from multiple flocks within each house. This is shown in Figure 2 (Davis et al., 2011).

## The environmental microbiome and low-level antimicrobial pressure from agriculture

The environment is both the main receptor for animal wastes containing AMR determinants, particularly those on MGEs (Heuer et al., 2011), and the locus of the natural resistome (D'Costa et al., 2006, 2007) and mobilome (Siefert, 2009; Gillings, 2013) which together constitute the genetic resources available to microbial communities for surviving antimicrobial stress and the genetic machinery to transfer these genetic resources among bacteria within and among different microbial communities. The environmental resistome, which encompasses both natural constituents as well as inputs from anthropogenic activities like agriculture, is relevant to human health as it can be a source of resistance determinants found in human pathogens, particularly through the mobilome (Forsberg et al., 2012; Perry and Wright, 2013).

There is ample evidence in the literature on the impacts of intensive food animal production on the occurrence of AMR in directly or indirectly affected environmental compartments (including soils, sediments, and water) (reviewed by Joseph et al., 2001; D'Costa et al., 2007; Ghosh and LaPara, 2007; Stine et al., 2007; Silbergeld et al., 2008; Graham et al., 2009a; Martinez, 2009; Knapp et al., 2010; Heuer et al., 2011; You et al., 2012, 2013; Gaze et al., 2013; Jones et al., 2013; Wei et al., 2013). Using quantitative methods such as real-time PCR and LC-MS/MS, correlations have been demonstrated between the occurrence/abundance of AMR genes and the extent of antimicrobial use or drug concentrations in animal husbandry environments (Smith et al., 2004; Peak et al., 2007; McKinney et al., 2010; Wu et al., 2010; Zhu et al., 2013). These relationships may involve multiple mechanisms including the simultaneous loading of both genes and drugs into the ecosystem and/or *in situ* selection for AMR in the environment due to inputs of antimicrobials, metals, and other residues. Until recently it was thought that the concentrations of antimicrobial compounds or their degradation products in the environment, which usually range from μg/kg to mg/kg in sediment or soil samples (Kemper, 2008), were not high enough to select for resistance. However, laboratory studies have demonstrated that low concentrations of antimicrobials in the same range are sufficient to select for resistance through several mechanisms (Kohanski et al., 2010; Gullberg et al., 2011). A study involving both field and laboratory research and coupled with modeling reported a selective and persistent effect of sulfadiazine in pig manure on resistance genes in soil microbiota (Heuer et al., 2008). Interactions between drugs (and their degradation products) and the soil microbiome are influenced by the physicochemical properties of each drug, which affect their potential bioavailability in the environment such as soils (Tolls, 2001; Hamscher et al., 2005; Kemper, 2008). Also antimicrobials undergo biotic/abiotic degradation in the environment (Sarmah et al., 2006), and only some of the degradation products exhibit antimicrobial potency (Halling-Sørensen et al., 2002). Data on the bioavailability of agricultural antimicrobials *in situ* are still missing (Heuer et al., 2011).

There are demonstrated links between agricultural antimicrobial use and expansion of the environmental resistome and mobilome (reviewed by Nandi et al., 2004; Ghosh and LaPara, 2007; Heuer et al., 2008, 2011; Zhang et al., 2009; Allen et al., 2010; Gaze et al., 2013; Zhu et al., 2013). A historical analysis of soil samples collected from 1940 to 2008 in the Netherlands found an exponential increase of AMR genes in agricultural soils (Knapp et al., 2010). Horizontal gene transfer largely contributes to the proliferation and persistence of AMR genes in the environment. After being introduced into the environment microbiome, animal-waste borne bacteria can transfer their AMR genes to indigenous bacteria through conjugation due to the frequent assembly of AMR genes on integrons, transposons and plasmids in the animal waste microbiome (Andrews et al., 2004; Nandi et al., 2004; Binh et al., 2008; Byrne-Bailey et al., 2011; Heuer et al., 2011; Zhu et al., 2013). This process can be stimulated by enhanced nutrient availability from animal wastes (van Elsas et al., 2003). Even after AMR bacteria of the animal gut microbiome died, their AMR genes can persist and be taken up by soil bacteria through transformation (Lorenz and Wackernagel, 1994) and thus proliferate in the environmental microbiome. Positive correlations have been observed between the concentration of antimicrobials and the abundance of MGEs carrying AMR genes in soil or water (Knapp et al., 2008; Zhu et al., 2013). But it is unclear whether this is due to enhanced horizontal gene transfer or selection on the recipient populations after transfer. Longitudinal and systemic studies on the environmental resistome and mobilome in response to agricultural stress are needed to assess its broad impacts on microbial ecosystems.

We have studied the presence of drugs, resistant bacteria, resistance genes, and resistance-encoding plasmids in soil microbiota near a waste storage site at a concentrated poultry production operation in the US in comparison to several sites within a protected state forest in the same region (You et al., 2012). Neither tetracycline nor chlortetracycline was detected in the forest soil samples, but measurable levels (<10 μg/kg) were detected in the farm samples from near the storage site. Farm samples also contained a high proportion of tetracycline resistant bacteria as compared to forest samples. Resistance genes (*tetM, O* and *ermA, B, C*) as well as plasmids containing *tetL* were only detected in farm soils. While *tetL* was found in both farm and forest samples, its prevalence was much higher in the former. In all of the perspectives, soil from a less waste-affected site at the same concentrated poultry production operation showed no significant difference from forest soil. These results are similar to those published on soils impacted by swine wastes (Agerso et al., 2006; Ghosh and LaPara, 2007). The results of our study suggested that resistance could persist within the soil microbiome. A study by Sengelov et al. (2003) reported persistence of resistant isolates for 300 days after application of swine wastes to soil. Another study by Ghosh and LaPara (2007) reported that bacterial resistance levels in soil with excessive application of swine manure were sustained for at least 18 months.

In summary, poultry and livestock wastes are an important anthropogenic source of antimicrobial pressure, at low levels, for the microbiomes of hosts and environments. The ecological and evolutionary events in the environmental microbiome in response to animal waste inputs include interactions between antimicrobials (also other chemicals as exemplified in the next paragraph) and the environmental microbiome; interactions between the waste microbiome and the environmental microbiome; and interactions between the environmental microbiome and resistance genes in waste. These interactions are relevant to the expansion and persistence of the resistome within the environmental microbiome, which may in turn expose human microbiomes through multiple pathways. Because of the complex nature of these interactions and the diversity of environmental microbiomes, studies at a systems level using advanced “omics” methods are in particular need.

## Interacting stressors in the gut and environmental microbiomes–antimicrobials and metals

Feeds for food animals are complex mixtures of natural products from both crop and animal sources, as well as recycled wastes and other additives (Sapkota et al., 2007). As a result, there are multiple potential stressors, in addition to GPAs, presented via feeds to food animal gut microbiomes. These include metals that are known to co-select for resistance in bacteria (Baker-Austin et al., 2006). For example, arsenicals are used as coccidiostats and growth promoters, copper and zinc are used as trace element supplements, mercury is present as a contaminant in fish meal (a major constituent of poultry feeds), as well as a range of metals in industrial waste byproducts permitted as additives to animal feeds (in the US). These metals are not metabolized (except in the case of arsenic, where chicken gut microbiota metabolize roxarsone, an organoarsenical, into the more toxic form of inorganic arsenic Stolz et al., 2007) and thus like antimicrobial drugs, they are excreted into wastes (Garbarino et al., 2003; Jackson et al., 2003) and transferred into soil through waste disposal (Gupta and Charles, 1999; Rutherford et al., 2003).

Previous microbiological research under both laboratory and field conditions have reported on interactions between these metals and antimicrobials in terms of co-selection for and co-transfer of resistance among bacteria via MGEs containing both metal and drug resistance genes (Bass et al., 1999; Baker-Austin et al., 2006; Singer et al., 2006; Stepanauskas et al., 2006; Wright, 2007; Tuckfield and McArthur, 2008; Novo et al., 2013). Significant positive correlations were observed between *tet* genes and several metals in a study of swine waste lagoons (McKinney et al., 2010), and between total AMR genes and copper in a study of manure, compost, and soils from swine farm (Zhu et al., 2013).

These interactions have extended to human health concerns. There is evidence that exposure of human hosts to mercury increases odds of their carrying antibiotic resistant *E. coli* (Skurnik et al., 2010). It has been known for some time that *tcrB*, a copper resistance gene, is transferrable and linked to genes encoding macrolide and glycopeptide resistance (Hasman and Aarestrup, 2002). This was recently confirmed independently by two research groups (Amachawadi et al., 2013; Silveira et al., 2014) who also showed that *tcrB* could be transferred by conjugation among *enterococci* from pigs, poultry, and cattle along with resistance genes for erythromycin, tetracycline, vancomycin, ampicillin, and gentamycin. Cavaco et al. (2011) reported that cadmium and zinc drive co-selection for methicillin resistance in *Staphylococcus aureus* through horizontal transfer of plasmids containing genes for both methicillin and metal resistance (*mec* and *czr*).

These findings indicate the need to consider interaction effects of antimicrobials and other stressors within agricultural settings in terms of driving AMR emergence and dissemination, particularly in light of the complex nature of manufactured feeds utilized in food animal production.

## Conclusions: Antimicrobial resistance is a form of environmental pollution

Resistance dissemination involves multiple microbiomes, each having a complex ensemble of microbes, particularly in the case of the environmental microbiome. These microbiomes are encountering diverse interacting stressors, and gene flow occurs through these microbiomes. Systems-biology approaches driven by “omics” methods (Raes and Bork, 2008) can improve our understanding of mechanisms of AMR development and persistence. Meanwhile, the “eco-health” perspective that takes into account linkages between ecosystems and health (Zinsstag et al., 2011) can improve our health practices, in particular those prior to human exposure.

An intriguing concept has been proposed, to consider AMR as environmental pollution, most comprehensively explained by Martinez (2009). There are some studies utilizing this concept to track AMR genes in landscapes, most frequently in studies of watersheds (Pei et al., 2006; Pruden et al., 2006). But this research, important as it is, still does not fully exploit the importance of the concept. If we think about “resistance” as a material thing, not just a behavior of bacteria, we can consider how this material behaves in environmental compartments such as water, soils, and sediments. Because of the importance of the environment as a locus for the emergence, persistence, and dissemination of AMR, particularly in relevance to low level AMR pressure derived from agriculture, this proposal merits further analysis at the theoretical level as well as more extensive field research in impacted environments.

In thinking about resistance as a pollutant, we can consider the approaches of environmental research in studying chemical pollutants. In many respects, resistance genes have the same properties that we consider in evaluating pollutants like pesticides.

Three characteristics are important in environmental health: hazard, persistence, and bioaccumulation. That is, does a substance in itself have properties that could seriously harm human health, does the substance remain unchanged in the environment without being broken down by natural processes, and finally is the substance taken up by organisms such that its levels increase over time in those species.

We can certainly say that an AMR gene is hazardous to human health because when it is present in a pathogenic bacterial cell or population it can result in failure of medical treatment of infection. Thus, resistance genes require incorporation into an organism to express their hazard, which is not so different from thinking the conversion of mercury into methyl mercury and its incorporation into fish. Low dose issues are important in understanding the hazards of agricultural use of antimicrobials since, similar to the hazards of some environmental chemicals, the pressure for selection for resistance may be more significant at lower doses (Vandenberg, 2014). This has been shown for failures of clinical antimicrobial treatment (Schentag et al., 2007).

The second characteristic of importance is persistence of resistance genes in the environment, about which we know relatively little. Resistance genes can survive in soils for long periods of time (Heuer et al., 2011). One recent study demonstrated that purified DNA from transplastomic plants encoding resistance to streptomycin could be detected as long as 4 years after being added into soils, and that these resistance genes could still be taken up by bacteria and incorporated into their chromosomal DNA for expression (Pontiroli et al., 2010). Persistence of resistant phenotypes is complex. There are both theoretical and empirical data to challenge the standard model of evolutionary selection for AMR or for susceptibility depending upon the presence or absence of antimicrobial stress (Andersson and Hughes, 2010). Theoretically, Levin and others have demonstrated that there are two evolutionary “choices” for resistant bacteria to adapt to the absence of stress when the expression of resistance exacts some cost to the bacterial populations in terms of physiological demands or reproductive rate (Levin et al., 2000; Rozen et al., 2007). One “choice” is reversion to susceptibility; the other choice is selection for a means to reduce this cost. Both choices involve mutation either back to the wild type gene or to a change in some other gene that is associated with increased Malthusian fitness. The second path may be a more adaptive strategy for microbial communities under continuous or periodic stress from antimicrobials, as in an environment with inputs of antimicrobial drugs from agriculture.

The third key characteristic of chemical pollutants in the environment in terms of raising alerts is bioaccumulation and biomagnification, or uptake and retention of chemicals in biota and increase in concentration within ecosystems through food chains. DNA (extracellular or intracellular) in the environment, like chemicals in the environment, can be accumulated by bacteria through transformation (Lorenz and Wackernagel, 1994) and on hierarchical compositions of MGEs through insertion (Frost et al., 2005). Biomagnification of a resistance gene occurs within microbiomes by the expansion of bacterial populations or MGEs carrying that gene. That is, (1) once a novel gene is taken up and incorporated into a bacterial genome, when that organism divides, its daughter cells each contain the new gene, which is a highly efficient process of increasing the total amount of that gene; or (2) once a novel gene is inserted in a MGE, when the MGE invades a bacterial community, diverse populations acquire the new gene through repeated infections.

## Research needs

From this brief review, we can identify some important research needs relevant to increasing our understanding of low dose antimicrobial exposures (including but not limited to GPAs in agriculture) in the context of microbial ecology and evolution. These questions in many cases can best answered with longitudinal studies using state of the art methods to interrogate the microbiome and its constituent elements such as the resistome and the mobilome. As noted by others, there is a critical lack of such studies at the systems level that permit examining associations between changes in the environmental resistome and well annotated changes in the drivers related to agricultural land use (Singer et al., 2006; Shade et al., 2013).

1. What does it mean to expose the microbiome to multiple antimicrobial and metal stressors at low doses?
2. What does it mean to stress the gut microbiome continuously over the lifetime and generations of food animals in terms of resistance and other changes at the microbiome level?
3. What events occur in food animal wastes in terms of expanding the resistome? What is the contribution of the continued presence of residual antimicrobials in wastes?
4. What events occur in soils where food animal wastes are repeatedly applied and in sediments impacted by agricultural runoff? What is the contribution of the continued presence of antimicrobials at low concentrations in soils and sediments?
5. How long is the persistence of resistant bacteria in animal wastes? How long is the persistence of resistance genes in the soil or sediment ecosystem?
6. How can we quantify the contribution of food animal production using GPAs to the expansion of the environmental and human host resistome/mobilome?
7. What are the major pathways from environmental compartments such as soils or sediments to humans; to what extent do these pathways involve passage through the microbiomes of wild or other domesticated animals?
8. What are the most effective and efficient methods for studying these events at the system level?

### Conflict of interest statement

The authors declare that the research was conducted in the absence of any commercial or financial relationships that could be construed as a potential conflict of interest.
